# Comparing the diagnostic accuracy of point-of-care lateral flow antigen testing for SARS-CoV-2 with RT-PCR in primary care (REAP-2)

**DOI:** 10.1016/j.eclinm.2021.101011

**Published:** 2021-07-13

**Authors:** Werner Leber, Oliver Lammel, Andrea Siebenhofer, Monika Redlberger-Fritz, Jasmina Panovska-Griffiths, Thomas Czypionka

**Affiliations:** a Centre for Primary Care, Wolfson Institute of Population Health, Barts School of Medicine and Dentistry, Queen Mary University of London, London, UK; b Practice Dr. Lammel, Ramsau am Dachstein, Austria; c Institute of General Practice and Evidence-based Health Services Research, Medical University of Graz, Graz, Austria; d Institute of General Practice, Goethe-University Frankfurt am Main, Frankfurt am Main, Germany; e Center for Virology, Medical University of Vienna, Vienna, Austria; f Big Data Institute, Li Ka Shing Centre for Health Information and Discovery, Nuffield Department for Medicine, University of Oxford, Oxford, UK; g The Queen's College, University of Oxford, Oxford, UK; h Institute for Global Health, University College London, London, UK; i Institute for Advanced Studies, Vienna, Austria; j London School of Economics and Political Science, London, UK

**Keywords:** Lateral flow antigen testing, Point-of-care testing, SARS-CoV-2, COVID-19, Primary care, Sensitivity, Specificity

## Abstract

**Background:**

Testing for COVID-19 with quantitative reverse transcriptase-polymerase chain reaction (RT-PCR) may result in delayed detection of disease. Antigen detection via lateral flow testing (LFT) is faster and amenable to population-wide testing strategies. Our study assesses the diagnostic accuracy of LFT compared to RT-PCR on the same primarycare patients in Austria.

**Methods:**

Patients with mild to moderate flu-like symptoms attending a general practice network in an Austrian district (October 22 to November 30, 2020) received clinical assessment including LFT. All suspected COVID-19 cases obtained additional RT-PCR and were divided into two groups: Group 1 (true reactive): suspected cases with reactive LFT and positive RT-PCR; and Group 2 (false non-reactive): suspected cases with a non-reactive LFT but positive RT-PCR.

**Findings:**

Of the 2,562 symptomatic patients, 1,037 were suspected of COVID-19 and 826 (79.7%) patients tested RT-PCR positive. Among patients with positive RT-PCR, 788/826 tested LFT reactive (Group 1) and 38 (4.6%) non-reactive (Group 2). Overall sensitivity was 95.4% (95%CI: [94%,96.8%]), specificity 89.1% (95%CI: [86.3%, 91.9%]), positive predictive value 97.3% (95%CI:[95.9%, 98.7%]) and negative predictive value 82.5% (95%CI:[79.8%, 85.2%]). Reactive LFT and positive RT-PCR were positively correlated (*r* = 0.968,95CI=[0.952,0.985] and κ=0.823, 95%CI=[0.773,0.866]). Reactive LFT was negatively correlated with Ct-value **(*****r*** **= -0.2999,*****p*** **< 0.001)** and pre-test symptom duration (*r* = -0.1299,*p* = 0.0043) while Ct-value was positively correlated with pre-test symptom duration (*r* = 0.3733),*p* < 0.001).

**Interpretation:**

We show that LFT is an accurate alternative to RT-PCR testing in primary care. We note the importance of administering LFT properly, here combined with clinical assessment in symptomatic patients.

**Funding:**

Thomas Czypionka received funding from the European Union's Horizon 2020 Research and Innovation Programe under the grant agreement No 101016233 (PERISCOPE). No further funding was available for this study.

**Research in context**

**Evidence before this study**

An effective test strategy is crucial for the detection of SARS-CoV-2 and lateral flow testing (LFT) may be a quick alternative to quantitative reverse transcriptase-polymerase chain reaction (RT-PCR). Evidence for effective symptomatic case detection with LFT at scale, in primary care and on the same patient cohort tested with PT-PCR is limited. After literature search, we found one comparable study that evaluated the accuracy of antigen testing using a single test device (Panbio COVID-19 Ag Rapid Test Device, Abbott), performed by staff experienced in point-of-care testing in nine primary healthcare centres in Spain.

**Added value of this study**

We provide evidence that LFT can accurately detect SARS-CoV-2 infection as an alternative to RT-PCR testing among symptomatic patients in a real-life primary care setting across a large geographical area. Our results suggest that LFT at the onset of symptoms and during early COVID-19 (when Ct-value is low) could be an accurate alternative to RT-PCR testing and a helpful strategy for curbing COVID-19 resurgences. Noting that in our study LFT was administered by self-taught clinicians and delivered at scale in primary care, we allude to the important aspect that population-wide LFT testing requires accurate administration of the test and this needs to be part of the planning strategy.

**Implications of all the available evidence**

This study has important implications for patients, general practice and public health, as once countries start easing lockdowns, early viral detection within community settings with population-wide testing will be an important part of the exit strategies alongside continual vaccination roll-out. We show that combined with immediate self-isolation, case notification and contact-tracing, population-wide testing with LFT can be part of an effective prevention and control strategy of the disease, via interruption of transmission chains. Therefore, our study supports efforts of population-wide LFT, but we note the importance of accurate administration of the tests, which in our case were conducted by self-trained primary care clinicians.

Alt-text: Unlabelled box

## 1 Introduction

Non-pharmaceutical interventions (NPIs) that include imposing severe social distancing restrictions (lockdown) on a national scale, effective implementation of national Test-Trace-Isolate (TTI) programmes with wide and mandatory use of face coverings, have been the main means of controlling the spread and preventing onward transmission of SARS-CoV-2 until the recent start of large-scale vaccination strategies. Effective TTI includes testing of symptomatic cases, tracing of their contacts and isolating those that test positive. While initial reports suggests effectiveness of vaccination in reducing hospitalisations and deaths from COVID-19 in age-cohorts that have been vaccinated, [1] its impact on reducing viral transmission is less clear [2]. In the meantime, when societies start to reopen with viral resurgence under control, testing interventions complementary to ongoing vaccination efforts, and mask wearing, will remain important in preventing onward transmission and future COVID-19 resurgence.

Testing is the first and crucial step of an effective TTI and a key in prevention and control of infectious disease. Reverse transcriptase-polymerase chain reaction (RT-PCR) is the gold standard for SARS-CoV-2 detection [3]. However, population-wide testing with RT-PCR is expensive, incurs a time delay in result return affecting the rates of contact tracing and adherence to self-isolation [4]. Furthermore, inclusion of asymptomatic people in population-wide testing has resulted in high false positive testing rates associated with low RT-PCR positivity [5]. False positive results may impact on personal lives, health systems and society [[5], [6], [7], [8]]. People may unnecessarily self-isolate, feel anxious and depressed, and incur financial loss due to absence from work; hospital appointments may be cancelled, and lockdown measures hardened and schools closed [5].

Rapid point-of-care antigen testing using lateral flow tests (LFTs) has been recommended as an alternative testing modality for symptomatic infection and in population-wide testing [3,9,10]. LFTs are much cheaper and can be produced in large quantities and deliver results much quicker: on site and in 15–30 min without the need for a laboratory. Previous studies have suggested that they may be less sensitive in detecting infections with low viral load, i.e. during early or late stage infections or when self-administered [3].

To date no comparative large-scale studies from primary care exist that compared the outcomes of both LFT and RT-PCR tests on the same patient cohort. As a number of countries are looking into using LFT in population-wide testing, comparing the outcomes of LFT and RT-PCR regimes, and evaluating the accuracy of implementing LFT in a general clinical setting such as primary care is fundamental to understanding the plausibility of this strategy. Our study assesses such diagnostic accuracy of LFT by comparing it to RT-PCR testing on the same primary care patients during the second COVID-19 wave in Austria.

General practice, which delivers access to care for large numbers of people, has been considered an important partner in supporting an effective TTI system [6]. Point-of-care testing is agreeable to patients and may improve engagement with their GP and self-management of their condition [11,12]. Patients report higher confidence in point-of-care testing if tests are accurate, [13] and if the tests are administered and results received directly from the GP [14]. Patients may feel less anxious with a shorter waiting time for the result, [15] and they may be more likely to engage with care when seeing the test result on the display screen of the test device [16]. Although major health system changes may be required to implement point-of-care testing in primary care, [17] it may improve diagnostic certainty and facilitate immediate clinical decision making [11]. So far, to our knowledge, only one study reported LFT among symptomatic patients in primary care [18]. In this study, LFT testing, when compared to RT-PCR, identified a small cohort of 43/412 (10.4%) people presenting within the first seven days of symptoms, on which the specificity and sensitivity were 100%, and 79.6% respectively.

Our study is set within a general practice network in the district of Liezen, Austria and includes a time period within the second COVID-19 epidemic wave (October 22 and November 30, 2020) in Austria. Following a relatively mild first wave of COVID-19 in spring 2020, Austria has faced a major second wave since September 2020, resulting in a nearly eight-fold increase in the number of daily cases compared to the initial outbreak. As of September 14, 2020, 23/46 (50%) practices, including 14 sentinel practices participating in the Austrian National Surveillance Network, [19] started offering LFT as a private service. Following our recent analysis of the early COVID-19 outbreak in a ski-resort in Austria (REAP-1), [20] on October 22, 2020, Austria published national guidelines for the implementation of antigen testing combined with clinical assessment in health care settings including general practice [21]. As of that day, antigen testing became available free of charge to symptomatic patients via a service contract with the Austrian Health Insurance.

Using the data from this general practice network, we aimed to quantify the diagnostic accuracy of LFT when compared to RT-PCR testing among the same patients presenting with mild to moderate flu-like symptoms to a network of general practitioners in the district of Liezen, Austria, in the midst of the second COVID-19 epidemic wave.

## 2 Methods

### 2.1 Setting

A network of 20 independent general practitioners in the district of Liezen (population 79,652), Austria, between October 22 and November 30, 2020. Data were available for this time period, and the lead clinician (OL) had access to all data. The study used anonymised data for which approval was granted by the Institute for Advanced Studies Research Ethics Committee, Austria (reference number: CASE002_2021_HEHP). Oral consent was obtained from all study participants.

### 2.2 Design

23 network practices were invited to participate in this prospective study. As per national guidelines, [21] patients with mild to moderate flu-like illness (including fever, cough, runny nose, sore throat, earache, diarrhea, general feeling of illness) received same-day appointment with a GP for clinical assessment including a nasopharyngeal swab for antigen testing. A subgroup of patients with non-reactive LFT, who presented with signs and symptoms suggesting of an alternative clinical diagnosis (e. g. glandular fever illness, bacterial tonsillitis and otitis media), were not suspected of COVID-19 and did not receive additional RT-PCR testing. This subgroup of patients was excluded from this study.

The remaining patients with suspected COVID-19 were tested with RT-PCR and categorised into four groups depending on their RT-PCR/LFT testing outcome. Patients testing RT-PCR positive were split into Groups 1 and 2, namely:

Group 1 (true reactive): Suspected COVID-19 cases with a reactive LFT, who tested RT-PCR positive.

Group 2 (false non-reactive): Suspected COVID-19 cases with a non-reactive LFT, who tested RT-PCR positive.

Subsets of Groups 1 and 2, for whom full data were available, were used in the descriptive, correlation and regression analyses. Patients testing RT-PCR negative were also split into those testing LFT reactive (false reactive) and LFT non-reactive (true non-reactive). Data from patients with negative RT-PCR, for whom CT-values were not available, were excluded from the regression analysis.

Suspected cases in Group 1 (reactive LFT and positive RT-PCR) were advised to immediately self-isolate and suspected cases in Group 2 (non-reactive LFT and positive RT-PCR) were advised to self-isolate till the return of the RT-PCR result (usually within 24 hours –48 hours). All patients received patient information sheets for self-care and were advised to call the practice or the national health hotline in case of clinical deterioration. For contact tracing, both the practitioners and the laboratories notified the local public health authority for any reactive LFT, and positive RT-PCR result respectively. People with false reactive LFT were released from self-isolation following receipt of a negative RT-PCR result, whilst those with non-reactive LFT continued to self-isolate for 10 days following a positive RT-PCR [21].

Five different commercial antigen test kits targeting the SARS-CoV-2 nucleocapsid antigen were used, including the Abbott Panbio Ag Rapid Test (sensitivity: 91.4%; specificity: 99.8%; https://www.abbott.co.uk), nal von minden NADAL COVID19-Ag Test (sensitivity: 97.6%; specificity: 99.9%; https://www.nal-vonminden.com), Roche SARS-CoV Rapid Antigen Test (sensitivity: 96.52%; specificity: 99.68%; https://www.roche.com/), Boson Rapid SARS-CoV-2 Antigen Test Card (sensitivity: 96.49%; specificity: 99.03%; https://www.bosonbio.com) and LumiraDX SARS-CoV-2 Antigen Test (sensitivity: 93.8%; specificity: 98.08%; https://www.lumiradx.com). All test kits meet the World Health organization (WHO) minimum performance criteria of ≥80% sensitivity of and ≥97% specificity [9]. Clinicians were advised to self-train on nasopharyngeal sampling using a YouTube video from Johns Hopkins University (https://youtu.be/DSrWjVyxEeg) and to follow the manufacturer's instructions on the use of test-kits.

RT-PCR testing was performed in three separate laboratories located in the cities of Vienna, Graz and Salzburg. The RT-PCR testing in Vienna was performed in scope of the routine surveillance at the Center for Virology, Medical University of Vienna. All three laboratories used Roche LightCycler (http://www.roche.com; Switzerland), and Graz additionally used the EURORealTime-SARS-CoV-2 test of EUROIMMUN (https://www.euroimmun.de/; Germany) according to the manufacturer's instructions. RT-PCR was considered positive at Ct-value of less than 40 as per the manufacturer's instructions, and ambiguous results were confirmed using RNA-dependent RNA polymerase (RdRP) gene detection (Vienna) and a BD MAX™ System using original SARS-CoV2 reagents of BD (https://www.bd.com/) (Graz).

### 2.3 Statistical analysis

Anonymised data on the number of LFT and RT-PCR tests across all practices were used to inform Groups 1 and 2. We report the number of cases detected with each test and evaluate the correlation of RT-PCR positivity and reactive LFT. Additionally, we quantify the diagnostic accuracy of LFT, by comparing it to RT-PCR via reported sensitivity (true-reactivity rate), specificity (true-non-reactivity rate) as well as the false-reactivity value, and the false-non-reactivity value. Additionally, correlation analysis across the entire dataset compared the correlation between the outcomes of LFT and RT-PCR testing estimating both the Pearson correlation coefficient (r) and the Cohen's kappa coefficient (κ) and their 95% Confidence Interval (95%CI). For a subset of the dataset, data was available across age, sex, pre-test duration of symptoms (defined as the period between onset of symptoms and antigen testing) and Ct-value (as a measure for inverse viral load) metrics in conjunction with the outcomes of the two tests. Logistic regression analysis quantified the association between reactive LFT with symptom duration and Ct-value. All analyses were completed using STATA 15. This study was reported using the Standards for Reporting of Diagnostic Accuracy Studies (STARD) guidelines [22].

## 3 Role of the funding source

The funding source did not have any role in study design, data collection, analysis, interpretation, summarizing the data or decision to submit the manuscript for publication.

## 4 Results

**Fig. 1:**
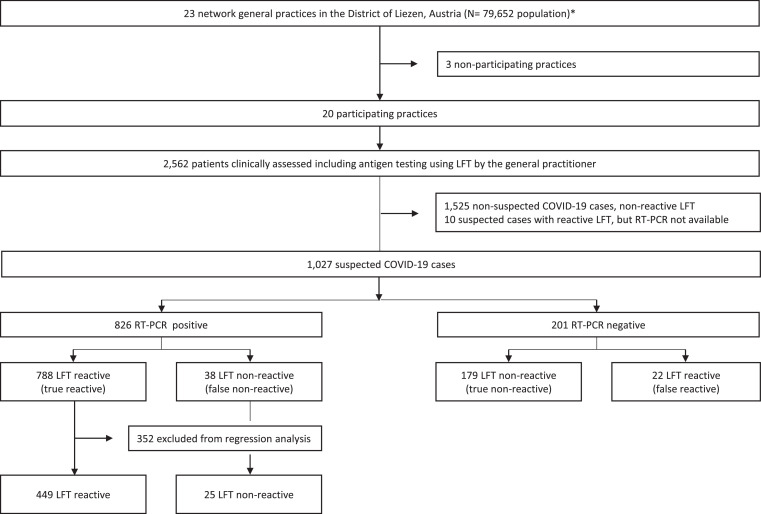
Flow-chart of the study: with the selection process, total numbers of included and excluded patients and details on the size of the groups based on the STARD guidelines[22]. *23/46 practices in the district of Liezen participated in the practice network.

Twenty of the 23 (87%) network practices participated in the study. Between October 22 and November 30, 2020, 2562 patients with mild to moderate flu-like illness were clinically assessed and tested with LFT across the 20 intervention practices (Fig. 1). 1525 patients, who tested LFT non-reactive and who presented with signs and symptoms suggesting a different type of infection, were not suspected of having COVID-19 and excluded from this analysis. The remaining 1037 patients with suspected COVID-19 infection had RT-PCR test with 826 (79.7%) testing RT-PCR positive and 201 (19.8%) testing RT-PCR negative (Table 2). For 10 (0.5%) patients RT-PCR results were not available and these 10 patients were excluded from further analysis.

Of those with negative RT-PCR, 179/201 tested LFT non-reactive (true non-reactive) and 22/201 (10.9%) tested reactive (false reactive) (Table 1), suggesting specificity of 89.1% (95%CI=[86.3%, 91.9%]).

**Table 1:** Sensitivity (true-positive), specificity (true-negative), positive predictive value (PPV) and negative predictive value (NPV), of lateral flow testing (LFT), when compared to reverse transcriptase-polymerase chain reaction (RT-PCR) on data from 1027 patients from a general practice network in Austria.

	RT-PCR negative	RT-PCR positive	Total tests	
LFT reactive	22	788	810	Positive Predictive Value 97.3%
LFT non-reactive	179	38	217	Negative Predictive Value 82.5%
Total tests	201	826	1027	
	Specificity 89.1%	Sensitivity 95.4%		

In patients testing RT-PCR positive, 788/826 tested LFT reactive (true reactive and within Group 1) and 38 (4.6%) tested LFT non-reactive (false non-reactive), suggesting sensitivity of 95.4%, 95%CI=[94%,96.8%]). Vice versa, in patients with reactive LFT tests, 788/810 tested RT-PCR positive (positive predictive value 97.3%,95% CI=[95.9%, 98.7%]), while in patients with non-reactive LFT, 179/217 tested RT-PCR negative (negative predictive value 82.5%,95% CI=[79.8%, 85.2%]) (Table 1).

Overall, for 1027 patients with both LFT and RT-PCR test outcomes, these were strongly correlated (*r* = 0.968, 95%CI=[0.952,0.985], *p* < 0.001; and κ=0.823, 95%CI=[0.773,0.866]).

Of the 1037 patients with RT-PCR test result, a total of 545 patients were dropped due to missing data on age (*N* = 344), sex (*N* = 344), duration of symptoms (*N* = 521) and Ct-value (*N* = 423); these included 352/826 patients with positive RT-PCR result, who were dropped from the regression analysis because of missing data on age, sex, duration of symptoms and Ct-value. Of the remaining 482 patients, 449 were LFT reactive and RT-PCR positive (Group 1), 25 were LFT non-reactive but RT-PCR positive (Group 2); the remaining 8 patients were LFT reactive but RT-PCR negative and hence had missing Ct-values. Consequentially, this small set of patients was also dropped from further analysis.

**Fig. 2:**
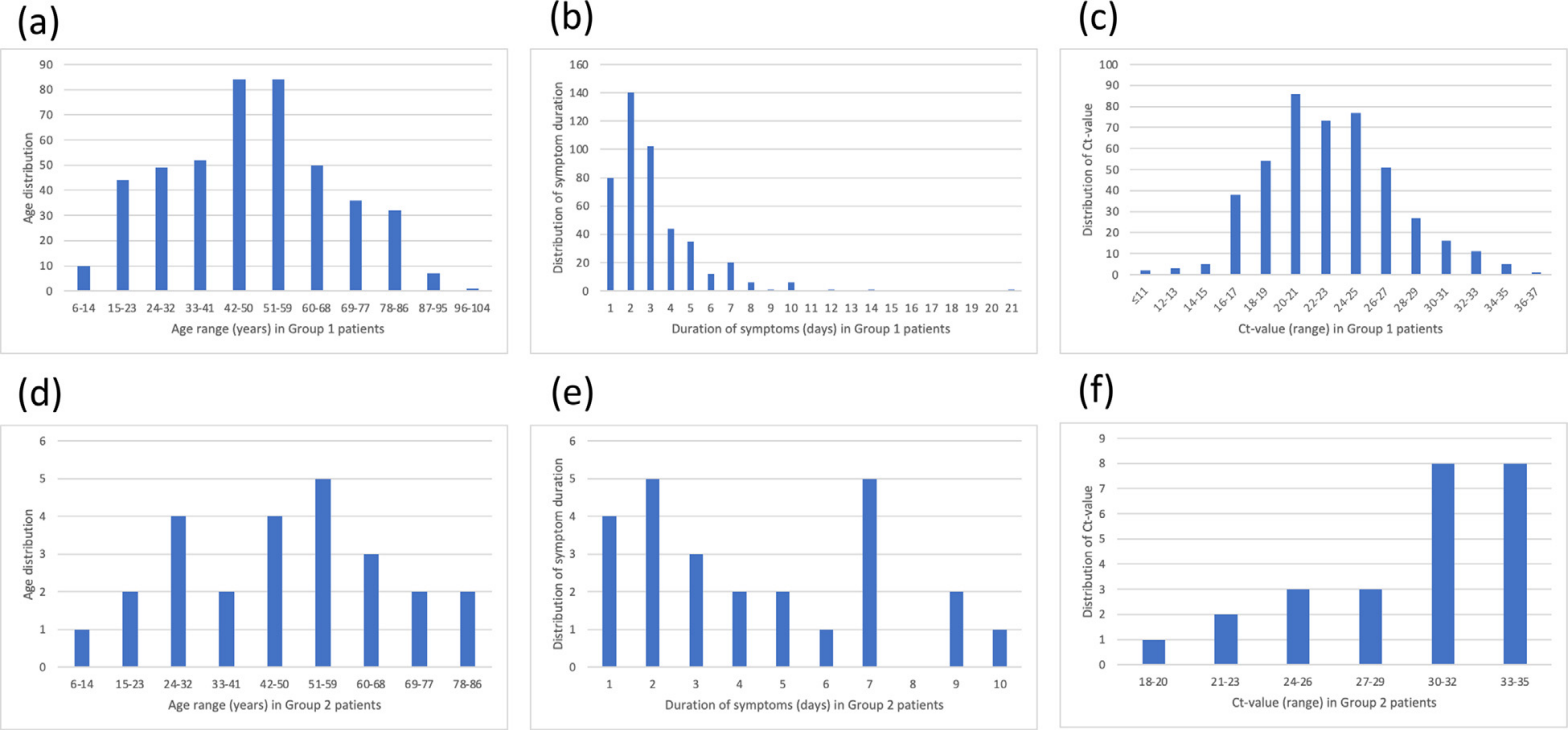
Distributions of age, duration of symptoms and Ct-values in patients who tested LFT reactive and RT-PCR positive (Group 1; (a-c)), and LFT non-reactive and RT-PCR positive (Group 2; (d-f)). The x-axis delineates the number of patients; and the y-axes delineate age in year ((a) and (d)), pre-test duration of symptoms ((b) and (e)), and Ct-value ((c) and (f)).

Descriptive statistics on the 474 patients with age, gender, duration of symptoms and Ct-values is shown in Table 2. Patients in Group 1 (true reactive) had shorter pre-test duration of symptoms (mean 3.1 days (range=[1,21])) than patients in Group 2 (false non-reactive) (4.4 days (range=[1,10])); lower Ct-value (mean 23 (range=[11,36]) vs 30 (range=[18,35])); but similar mean age (49 years (range=[11,83]) vs. 48 years (range=[6,102])). Furthermore, despite a difference in the data size for Groups 1 and 2, there was a notable difference in the distributions across these variables between both groups (Fig. 2).

**Table 2:** Baseline characteristics of the study participants, and LFT and RT-PCR testing results among the 474 patients for whom we have full data on age, sex, Ct-value and outcome of both tests. Legend: LFT, lateral flow testing; RT-PCR, reverse transcriptase-polymerase chain reaction; Ct, cycle threshold value.

	Group 1: LFT reactive (*n* = 449)	Group 2: LFT non-reactive (*n* = 25)	RCT-PCR positive (*n* = 474)
Age, mean (range)			
<18	15(6–14)	14(11–18)	15(6–18)
18–34	34(18–34)	26(20–32)	26(18–34)
35–49	44(35–49)	44(37–49)	44(35–49)
50–59	54.5(50–59)	54(50–59)	54(50–59)
60–69	64.5(60–69)	64(60–64)	63(60–69)
70–79	76(70–79)	72(71–73)	75(70–79)
≥80	92(80–102)	82(81–83)	84(80–102)
Females (%)	253(56.3%)	13(52%)	266(56.12%)
Pre-test duration of symptoms, mean days (range)			
1–3	2.1(1–3)	1.92 (1–3)	2.063(1–3)
4–7	4.7(4–6)	4.80(4–6)	4.64(4–6)
>7	8.4(7–21)	7.88(7–10)	8.311(7–21)
Pre-test duration of symptoms, number of patients (%)			
1–3	322(71.6%)	12(48%)	334(70.5%)
4–7	91(20.3)	5(20%)	96(20.3%)
>7	36(8.0%)	8(32%)	44(9.2%)
Ct-value, mean (range)			
<30	21.9(10.9–29.9)	24.5(18–28)	22(10.9–29.9)
≥30	32.2(30–36)	32.3(30–35)	32(30–36)
Ct-value, number of patients (%)			
<30	423(94.2%)	9(36%)	431(90.9%)
≥30	26(5.8%)	16(64%)	43(9.1%)

### 4.1 Intercorrelation analysis

Reactive LFT was negatively and significantly correlated with Ct-value (*r* = −0.2999, *p* < 0.001) and duration of symptoms (*r* = −0.1299, *p* = 0.0043), but the association was not statistically significant with sex (*r* = −0.0199, *r* = 0.6628) and with age (*r* = 0.0099, *p* = 0.8292). Ct-value was positively correlated with symptoms (*r* = 0.3733), *p* < 0.001), but not with age (*r* = 0.0307, *p* = 0.505) or sex (*r* = 0.0778, *p* = 0.0907).

### 4.2 Logistic regression

Univariate logistic regression analysis suggests that reactive LFT is strongly associated with Ct-value <30 ((OR = 0.0345, 95%CI=[0.0134,0.0891], *p* < 0.001) and duration of symptoms of 1–3 days (OR=0.425, 95%CI=[0.235,0.769], *p* = 0.005).

## 5 Discussion

Evidence on the performance of SARS-CoV-2 LFT delivered at scale and compared to RT-PCR on the same primary care cohort is lacking. This study demonstrates that using LFTs for people presenting with mild to moderate flu-like symptoms can reliably and accurately detect SARS-CoV-2 and is comparable to RT-PCR detection. Across a large network of 20 general practices, RT-PCR positivity rate among suspected cases was high at 79.7%; overall LFT sensitivity was 95.4%; and specificity was 89.1%; with high positive predictive (97.3%) and negative predictive values (82.5%). Both shorter duration and symptoms and lower Ct-value were significantly associated with reactive LFT. Combined with clinical assessment, LFT enables high yield and accurate antigen detection of COVID-19 infectivity among symptomatic patients in primary care.

Our findings are consistent with studies conducted in hospital and community (drive through and walk-in centers) and primary care testing sites, linking sensitivity of antigen testing with shorter duration of symptoms and low Ct-value. In hospital settings, the Foundation for Innovative New Diagnostics (FIND) reported higher sensitivity with shorter duration of symptoms (≤7 days) compared to overall sensitivity for both the Abbott (Germany 80% to Brazil 90.7% vs. 76.8% to 88.7%) [23] and the Roche (Switzerland 85.6% to Germany 90.8% vs. 85.5 vs. 86.8%) [24] platforms, and sensitivity was highest at Ct-value ≤25 for both the Abbott (96.8% vs. 95.8%) and Roche (100% to 95.9%) systems. Similarly, a study conducted in two community testing sites also demonstrated higher sensitivity at low Ct-value <32 compared to overall sensitivity in both Utrecht (Netherlands) (95.2% vs 72.6%) and Aruba (98% vs. 81%) respectively [25]. A study conducted under “real-life” conditions among patients presenting within seven days of symptom onset in primary care demonstrated 100% sensitivity for Ct<25, compared to the overall sensitivity of 79.6% for the Abbott Panbio test kit [18]. In addition, in terms of sensitivity, nasopharyngeal sampling by clinicians may be superior to supervised self-swabbing. Sensitivity was higher when conducted by lab scientists (70.2%) or trained health care workers (73%) when compared to self-swabbing by self-trained members of the public (57.5%) [26]. To rule out infection in a high prevalence setting, high test sensitivity (true positive value) is more important than high specificity (true negative value) as a highly sensitive test means there are fewer false negative results and thus fewer cases of infection are missed [27]. In our study, sensitivity was 95.4% suggesting that LFT could rule out infection with more than 95% confidence among those 4.9% of suspected cases who tested LFT non-reactive. Furthermore, 16/25 of suspected cases with false non-reactive LFT (64%) had CT-value ≥30, suggesting that these cases were likely no longer infectious at the time of testing.

The specificity (89,1%) in our study is lower than any value of clinically validated tests (93.1 to 100%) reported to the ECDC [3]. Our low specificity likely reflects real-world performance of LFT and may represent faulty test kits, incorrect test kit storage [28] or test handling such as insufficient specimen collection or incorrect timing of incubation of the test [10]. In addition, a false non-reactive LFT might have resulted from a false negative RT-PCR test result, [29] and false negative test results due to poor sample quality or improper transport to remote laboratories cannot be excluded [30,31]. The only comparable study from primary care, which achieved high specificity (100%), was conducted in nine primary care centres by nurses with prior experience in point-of-care testing and one single test kit from Abbott Panbio (personal communication) [18]. In contrast, in our study five different LFT test kits were used across a large number of practices, and lack of coordination in implementing testing and of training for clinicians in interpreting the test results may have impacted on the accuracy of the test [32].

In line with these studies, the optimal performance of LFT could occur among symptomatic patients presenting early and receiving nasopharyngeal swabs from clinical staff in community settings. LFT of symptomatic patients in primary care should supplement existing population-wide testing strategies and may assist rapid and accurate testing in other settings as part of the gradual reopening of schools and society. However, training and quality assurance of test operators and co-ordination of services may be required to optimise test outcomes [32].

Our study has many strengths. It was conducted in a wide geographical area and across a large network of practices (*N* = 20), including 14 (70%) sentinel practices participating in the Austrian Influenza Surveillance Network [19]. Data were generated using five different types of antigen testing platforms and across three laboratories located in different parts of Austria, reflecting the realities of implementing a local service across a wide geographic region. The practice network was formed in response to our initial study, [20] a looming second wave, and national recommendation for implementation of antigen testing in health care settings, including general practice [21]. Both the lead researcher (WL) and statistician (JPG) are routinely involved in service evaluations [20,33]. Data were collected prospectively using a standard clinical questionnaire and the lead clinician (OL) collated and cross-linked data in a single database. Our protocol recommending self-isolation for all suspected cases (irrespective of the LFT result) mitigated both the risk of unnecessary prolonged self-isolation among patients with false reactive LFT and the risk of infection to others from patients with a false non-reactive result [29].

Our study has some limitations. Practices independently procured both LFT kits and RT-PCR testing contracts with one university and two private laboratories preventing the determination of the accuracy of individual test systems used. Clinicians were self-taught and used five different types of test kits across a large number of practices. Ct-values can vary between laboratories RT-PCR platforms and may not be directly comparable [34]. Finally, due to the lack of a primary care patient register in Austria, we were unable to calculate the rate of LFT testing across the practices.

Importantly, our study is the first study to demonstrate that point-of-care antigen testing using LFT combined with clinical assessment of symptomatic patients can rapidly and accurately detect SARS-CoV-2 infection in primary care. Our study has important implications for patients, general practice and public health. Symptomatic patients are likely to benefit from both access to testing in a familiar primary care setting and when they are most infectious, same-day clinical assessment with immediate notification of test results, and opportunity for early self-isolation for those testing LFT reactive. Prompt public health notification of reactive LFT results may also speed up the process of contact tracing. LFT testing delivered through a clinical network enables intervention implementation across a wide geographical area, knowledge exchange between practitioners, and better integration with laboratory and public health services. As the vaccination roll out continues and many countries start easing lockdown, early viral detection within community settings will form an important adjunct to national TTI strategies. Immune evasion and emergence of new virus variants may produce future outbreaks. Given the acceptability for point of care testing among patients, early viral detection using LFT, followed by immediate case isolation and effective contact tracing, would assist in detecting outbreaks early, hence promptly interrupting transmission chains. Since publication of our first study (REAP-1)[20], and motivated by its results, Austria has increased its sentinel sites from 91 to 231 general and pediatric practices including a majority (70%) of practices participating in this study, indicating that primary care has become an important strategic partner in implementing the national surveillance system. Future recommendations for research should include an evaluation of the impact of REAP-2 on regional RT-PCR positivity rates and modelling of antigen testing with LFT at scale vs. different levels of lockdown and vaccination roll-out strategies.

In summary, we show that symptomatic antigen testing for SARS-CoV-2 with LFT delivered at scale in a general clinical setting such as primary care as early as onset symptom, and when viral load is high and Ct-value is low, can rapidly and accurately detect early COVID-19 among patients presenting with flu-like illness and can be a plausible alternative to RT-PCR. Implementation of LFT should be accompanied by standardised training for test operators, quality assurance of testing, and coordination of services.

## Authors’ contributions

OL set up the general practice network and promoted the intervention among participating network practices. OL, WL, TC, AS, MRF, and JGP contributed to the design of the study. OL collated all patient data. WL wrote the study protocol, TC submitted the ethics application, and MRF provided RT-PCR data. JPG and WL conducted the statistical analysis. WL, JPG and AS wrote the manuscript with contributions from OL, MRF, and TC. All authors read and approved the final version.

## Data sharing statement

The datasets used and analysed during the current study are available from the corresponding author on reasonable request.

## Funding

Thomas Czypionka received funding from the European Union's 10.13039/100010661Horizon 2020 Research and Innovation Programe under the grant agreement No 101016233 (PERISCOPE). No further funding was available for this study.

## Declaration of Competing Interest

None declared.
